# 2 + 1 dimensional de Sitter universe emerging from the gauge structure of a nonlinear quantum system

**DOI:** 10.1038/s41598-017-08183-8

**Published:** 2017-08-29

**Authors:** Chon-Fai Kam, Ren-Bao Liu

**Affiliations:** Department of Physics & Centre for Quantum Coherence, The Chinese University of Hong Kong, Shatin, New Territories, Hong Kong China

## Abstract

Berry phases and gauge structures are fundamental quantum phenomena. In linear quantum mechanics the gauge field in parameter space presents monopole singularities where the energy levels become degenerate. In nonlinear quantum mechanics, which is an effective theory of interacting quantum systems, there can be phase transitions and hence critical surfaces in the parameter space. We find that these critical surfaces result in a new type of gauge field singularity, namely, a conic singularity that resembles the big bang of a 2 + 1 dimensional de Sitter universe, with the fundamental frequency of Bogoliubov excitations acting as the cosmic scale, and mode softening at the critical surface, where the fundamental frequency vanishes, causing a causal singularity. Such conic singularity may be observed in various systems such as Bose-Einstein condensates and molecular magnets. This finding offers a new approach to quantum simulation of fundamental physics.

## Introduction

Quantum phases are essential in many aspects of quantum physics, many-body physics, and quantum field theories. A spectacular feature of quantum phases is the appearance of geometric phases in adiabatic processes. In 1984, Berry discovered that in addition to the conventional dynamical phase, a geometric phase shift of a wave function is induced by a cyclic adiabatic change of parameters^1^, which depends only on the shape of the cycle in parameter space. Specifically, geometric phases arise from the overlap of coherent states^2^ along a closed path in the space of quantum states and play an indispensable role in the development of gauge field theories^3^. Almost at the same time as Berry’s discovery of Berry’s phases, Simon recognized from discussions on the relationship between geometric phases and Chern integers^4^ that the geometric phase is precisely the holonomy in fiber bundle theory^5^ — while the wave function is single-valued in the space of quantum states, it can be multi-valued around a cycle in the space of parameters. Following insights from Berry and Simon, Hannay^6, 7^ showed that the shift of a classical phase angle in response to a cyclic adiabatic change of parameters is also a manifestation of the holonomy effect. It soon became clear that geometric phases are powerful tools for investigating a wide variety of intriguing properties of gauge field theories and are the basis of a broad range of phenomena and applications, such as quantum Hall effects^8^, topological insulators and superconductors^9, 10^, artificial gauge fields in cold atomic gases^11^, holonomic quantum computation^12, 13^, and quantum interference effects in molecular magnets^14, 15^.

**Figure 1:**
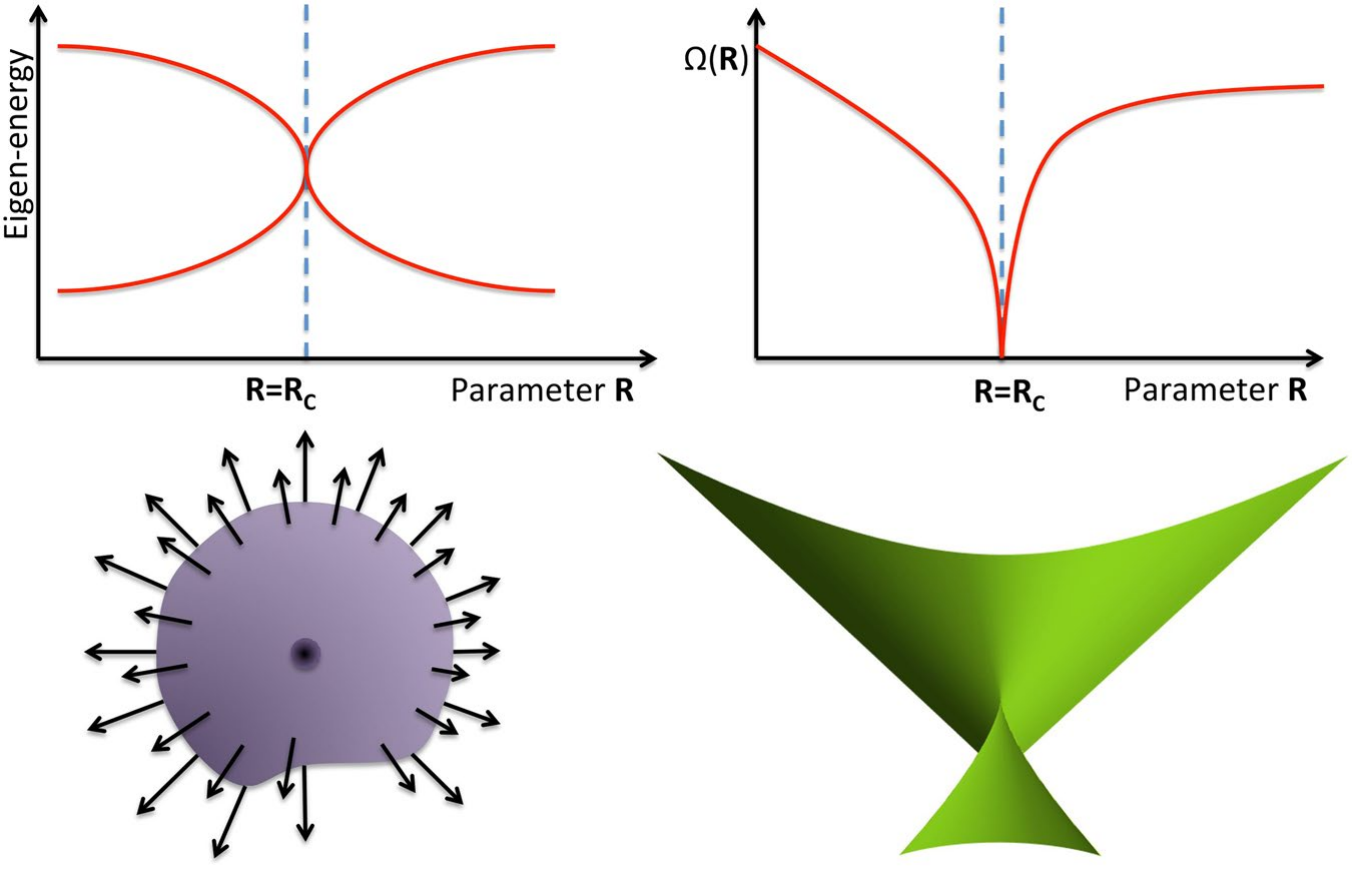
Gauge structure anomalies in linear quantum systems versus those in nonlinear quantum systems. In linear quantum systems, the degeneracy of energy levels is associated with a magnetic monopole in the parameter space; by contrast, in nonlinear quantum systems, the vanishing of the fundamental frequency of the Bogoliubov excitations is associated with a critical surface in the parameter space. Here the critical surface is shown to have a swallowtail singularity at the origin.

A fundamental feature of geometric phases is the emergence of magnetic monopole singularity^16, 17^ associated with energy level degeneracy^18, 19^. In a general evolution of a quantum state, the geometric phase is characterized by a gauge invariant field, namely, the Berry curvature. The Berry curvature diverges at degeneracy points, and can be regarded as an effective magnetic field with the point of degeneracy acting as its source, that is, a magnetic monopole in the parameter space (see Fig. 1). The existence of magnetic monopoles reflects the global nature of the parameter space and is invariant under local perturbations to the spectra^20^.

**Figure 2:**
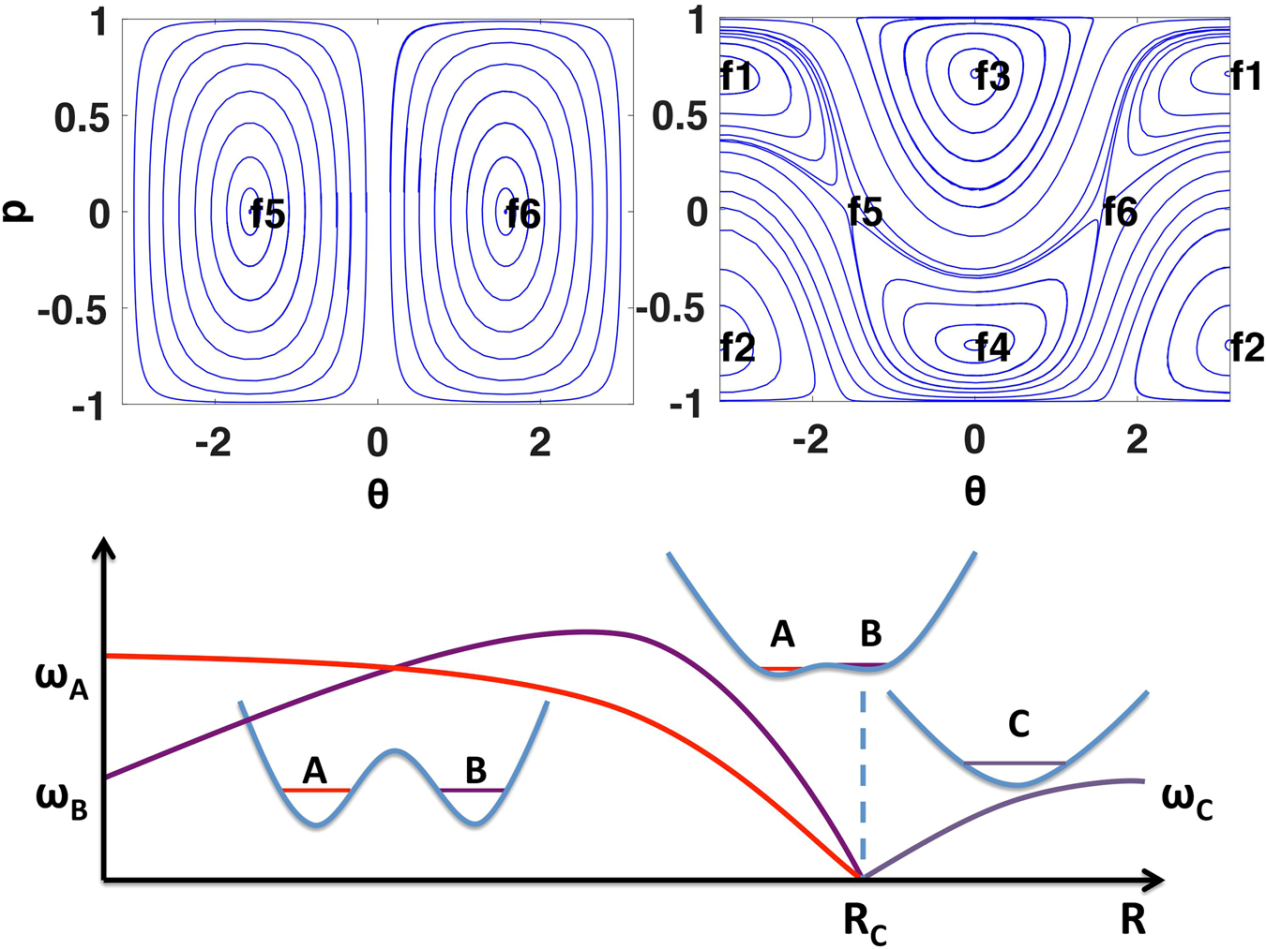
Phase transitions of a nonlinear quantum system. Here R_c_ is a critical value of R. For R < R_c_, there are two Bogoliubov modes A and B with fundamental frequencies ω_A_(R) and ω_B_(R), respectively. The two fundamental frequencies drop to zero at R = R_c_, which induces the mode softening. For R > R_c_, the two modes merge into a new Bogoliubov mode C with fundamental frequency ω_C_(R). Here, the phase space portrait is obtained from the two mode-model in Eq. (3) with ${\rm{\Delta }}=\epsilon =0$, where the fixed points f_5_ and f_6_ correspond to $(\bar{p},\bar{\theta })=(0,\pm \pi /2)$. Surrounding the fixed points f_5_ and f_6_, there are two modes A and B with finite fundamental frequencies $\sqrt{\alpha \gamma -{\beta }^{2}}$, both of which drop to zero at *αγ* − *β* ^2^ = 0.

Geometric quantum phases of interacting many-body systems are particularly interesting for their exotic features^21–23^. While quantum systems are intrinsically linear due to the superposition principle, nonlinear quantum mechanics, as an effective theory derived from a mean field treatment of interacting many-body quantum systems, can be used to describe condensates of interacting bosons^24^ and quantum nanomagnets^25^. For example, the dynamics of the order parameter of interacting bosons can be effectively described by a nonlinear Schrödinger equation^26, 27^. In contrast with linear quantum systems, the superposition principle is no longer valid in nonlinear systems^28^. The definition of geometric phases of nonlinear quantum systems is subtle due to the breakdown of the linear superposition principle. In particular, quantum phase transitions^29^ can occur in nonlinear quantum systems with a change of external parameters. At the critical surfaces in parameter spaces where quantum phase transitions occur, the order parameter vanishes and so the adiabatic phases of the order parameter are expected to present singularities (see Fig. 2). The magnetic monopole paradigm is insufficient to reflect such singularity associated with quantum critical phenomena^30^. In nonlinear quantum systems, the central concept involved is elementary Bogoliubov excitations^21, 22^, whose fundamental frequencies vary with external parameters. The fundamental frequencies of the Bogoliubov excitations vanish at the critical surfaces and the divergence of the time scale, that is, the inverse of the fundamental frequency at the critical surface, indicates the emergence of singularity in the adiabatic evolution in parameter space (see Fig. 2).

The canonical formalism of quantum mechanics has been introduced to formulate the geometric phases of nonlinear quantum systems so as to overcome the difficulties arising from the lack of superposition principle^31^. In this paper we will employ this formalism to explore the connections between geometric phase singularities and quantum critical phenomena. In the canonical formalism, the wave function is regarded as a classical field which can be used to describe the order parameter of a Bose-Einstein condensate^32^ or a collective spin system^33^. The geometric phases of quantum evolution are then formulated as Hannay phases^6, 7^ of classical harmonic oscillators that correspond to the Bogoliubov excitations of quantum many-body systems^21, 22, 31^. For a discrete system with mode index *k*, such as a Bose-Einstein condensate in a double-well trap, the time evolution of the wave function is governed by a set of coupled equations^28^

1 $$i\frac{d{\psi }_{k}}{dt}=\frac{\partial }{\partial {\psi }_{k}^{\ast }}H(\psi ,{\psi }^{\ast },{\bf{R}}).$$

Here the Hamiltonian *H* is a real function of *ψ* and *ψ* ^*^, and depends on some external parameters R. For instance, the coherent atomic tunneling between two Bose-Einstein condensates confined in a double-well trapping potential is described by the nonlinear Hamiltonian

$$H=\epsilon ({|{\psi }_{1}|}^{2}-{|{\psi }_{2}|}^{2})+{\rm{\Delta }}({\psi }_{1}^{\ast }{\psi }_{2}+{\psi }_{1}{\psi }_{2}^{\ast })+\frac{\gamma }{2}{({|{\psi }_{1}|}^{2}-{|{\psi }_{2}|}^{2})}^{2},$$

where $\epsilon$ is the difference between the single-mode energies, Δ is the Josephson tunneling rate, and *γ* is the nonlinear parameter proportional to the overlap of the spatial wave functions localized in each potential well. In the canonical formalism of quantum mechanics, linear or nonlinear, the amplitude ${p}_{k}={|{\psi }_{k}|}^{2}$ and the phase ${\theta }_{k}={\rm{\arg }}\,{\psi }_{k}$ of the wave amplitudes form a pair of canonical coordinates, and the time evolution of the wave amplitude can be mapped to the corresponding classical dynamics. In this simple double-well model, the classical Hamiltonian has the form^21^

2 $$H=\epsilon p+\frac{\gamma }{2}{p}^{2}+{\rm{\Delta }}\sqrt{1-{p}^{2}}\,\cos \,\theta ,$$

where *p* = *p*_1_ − *p*_2_ is the population imbalance between the two wells and *θ* = *θ*_2_ − *θ*_1_ is the relative phase between the two macroscopic wave functions. Notice that the global phase *λ* = *θ*_1_ + *θ*_2_ is absent in the classical Hamiltonian, as a consequence of the conservation of total population *p*_1_ + *p*_2_. For the case of a symmetric well, the Hamiltonian describes a classical non-rigid pendulum of tilt angle *θ* and a length proportional to $\sqrt{1-{p}^{2}}$.

In the canonical formalism of quantum mechanics, the linearized dynamics near fixed points are characterized by the fundamental frequencies of vibrations which correspond to the Bogoliubov excitations from the ground states. In the absence of nonlinearity, the fundamental frequencies are equivalent to the energy level spacings. However, the appearance of nonlinearity causes a bifurcation of the classical dynamics, which results in a qualitative change in the topology of the trajectories in phase space. In particular, the bifurcation of the dynamics in phase space implies the existence of quantum criticality in the original quantum system. Figure 2 illustrates a sample system, for example, of a Bose–Einstein condensate in a double-well trapping potential, in which a variation of control parameters results in a change of the topological type of the dynamics. In the simplest case, the topological type only depends on a single control parameter *R*. For *R* < *R*_*C*_, there are two distinct Bogoliubov modes *A* and *B* with different fundamental frequencies *ω*_*A*_(*R*) and *ω*_*B*_(*R*) respectively. At the critical point *R* = *R*_*C*_, the two Bogoliubov modes experience mode softening with their fundamental frequencies both dropping to zero. For *R* > *R*_*C*_, the two discrete modes merge into a new Bogoliubov mode *C*. In this regard, the quantum criticality in a nonlinear quantum system is not induced by the degeneracy of energy levels, but is rather caused by the softening of the Bogoliubov modes. Such an analysis can be applied to cases where there is more than one control parameter and to cases where there are different numbers of Bogoliubov modes before and after a given phase transition. When the control parameters are adiabatically varying, the disappearance of Bogoliubov modes and emergence of new ones cause quantum criticality at a critical surface in the parameter space. Near the critical surface the system exhibits mode softening, that is, the oscillation has an infinite period and all the energy levels collapse. In view of these facts, the oscillation period could be regarded as the clock of the system as it determines the characteristic time scale of the dynamics. This observation leads to a natural description of geometric phases in the presence of quantum critical phenomena not based on the traditional magnetic monopole paradigm, but in terms of the evolution of spacetime in classical relativity.

**Figure 3:**
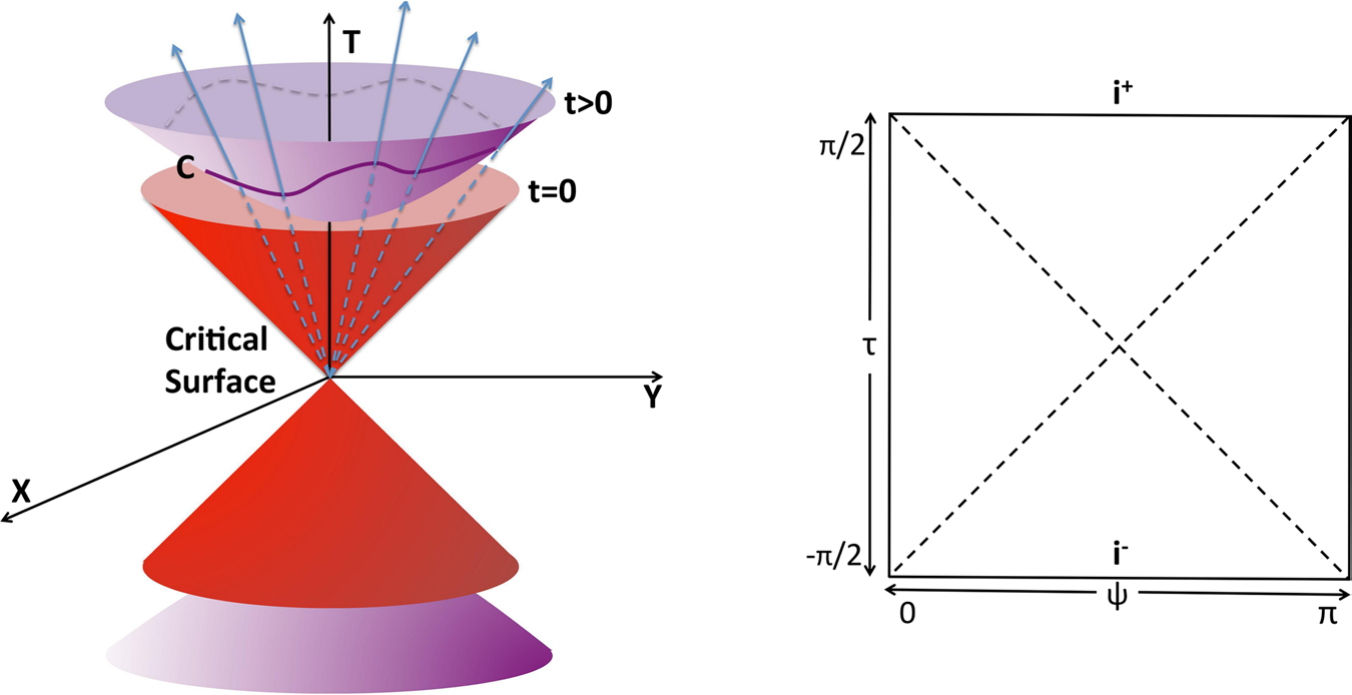
Conic singularity of a nonlinear quantum system. (**a**) T, X and Y denote the external parameters of the Hamiltonian. The red cone locates the critical surface in parameter space where the proper distance vanishes. C denotes a closed curve and the classical adiabatic angle is the area bound by C on the unit hyperboloid. (**b**) The Penrose diagram shows the causal structure of the de Sitter space, which is obtained by the usage of conformal coordinates (*τ*,*ψ*,*φ*) defined by $T=\tan \,\tau$, $r={\cos }^{-1}\,\tau \,\sin \,\psi$ and $Z={\cos }^{-1}\,\tau \,\cos \,\psi$, where $X=r\,\cos \,\varphi$ and $Y=r\,\cos \,\varphi$. In conformal coordinates, the metric has the form $d{s}^{2}={\cos }^{-2}\,\tau \,(-d{\tau }^{2}+d{\psi }^{2}+{\sin }^{2}\,\psi d{\varphi }^{2})$. Each point in the plot represents a circle and the left and right hand sides correspond to the north *ψ* = 0 and south *ψ* = *π* poles respectively. The upper and lower sides labelled *i* ^+^ and *i* ^−^ denote the infinite past and infinite future respectively.

In this work, we report our discovery that the classical geometric phase of a generalized harmonic oscillator that corresponds to the Bogoliubov mode of a nonlinear quantum system can be explained by the global geometry of a de Sitter universe^34^ described qualitatively by the Friedmann-Lemaître-Robertson-Walker metric^35^. In our method, the fundamental frequency of the oscillation near the critical surface plays the role of the cosmic scale factor^34, 35^, and the classical geometric phase is an integral of a differential 2-form that exhibits a conic singularity similar to the casual singularity at the big bang^36^ of the de Sitter universe (see Fig. 3).

## Results

We consider the Hamiltonian of a nonlinear quantum system with two wave amplitudes *ψ*_1_ and *ψ*_2_, which up to fourth order can be written as

3 $$\begin{array}{rcl}H & = & {\rm{\Delta }}({\psi }_{1}^{\ast }{\psi }_{2}+{\psi }_{1}{\psi }_{2}^{\ast })+\epsilon ({|{\psi }_{1}|}^{2}-{|{\psi }_{2}|}^{2})+\frac{\alpha }{2}{({\psi }_{1}^{\ast }{\psi }_{2}+{\psi }_{1}{\psi }_{2}^{\ast })}^{2}\\  &  & +\beta ({\psi }_{1}^{\ast }{\psi }_{2}+{\psi }_{1}{\psi }_{2}^{\ast })\,({|{\psi }_{1}|}^{2}-{|{\psi }_{2}|}^{2})+\frac{\gamma }{2}{({|{\psi }_{1}|}^{2}-{|{\psi }_{2}|}^{2})}^{2},\end{array}$$

where Δ, $\epsilon$, *α*, *β* and *γ* are time-dependent external parameters that are characteristics of the system (see SI, Note 1 for a physical realization of the Hamiltonian in a double-well Bose-Einstein condensate). As the Hamiltonian is invariant under global phase transformation, the total probability of the two wave amplitudes is conserved, ${|{\psi }_{1}|}^{2}+{|{\psi }_{2}|}^{2}=1$, and we can make the substitution ${\psi }_{k}=\sqrt{{p}_{k}}{e}^{i{\theta }_{k}}$, which yields *p*_1_ + *p*_2_ = 1. If we define *p* = *p*_1_ − *p*_2_ and *θ* = *θ*_2_ − *θ*_1_, the Hamiltonian becomes

$$H=\epsilon p+\frac{\gamma }{2}{p}^{2}+({\rm{\Delta }}+\beta p)\sqrt{1-{p}^{2}}\,\cos \,\theta +\frac{\alpha }{2}(1-{p}^{2})\,{\cos }^{2}\,\theta .$$

In the special case where ${\rm{\Delta }}=\epsilon =0$, $\bar{p}=0$ and $\bar{\theta }=\pi /2$ is a fixed point of the classical dynamics described by the canonical formalism of the nonlinear quantum system. Near the fixed point, the linearized Hamiltonian has the form of a generalized harmonic oscillator with time-dependent coefficients

4 $$H\approx (\alpha (t){\theta }^{2}+2\beta (t)p\theta +\gamma (t){p}^{2})/2.$$

For *αγ* > *β* ^2^, the Hamiltonian describes stable oscillations with elliptical trajectories in phase plane; for *αγ* < *β* ^2^, the origin is a saddle fixed point, and the trajectory contours become hyperbolae in phase space. For a given energy *E*, the area of the ellipse is 2*πE*/(*αγ* − *β* ^2^)^1/2^, a point on the ellipse is denoted by an angle variable Θ, and the frequency of oscillation is $\omega =\sqrt{\alpha \gamma -{\beta }^{2}}$. Thus, *αγ* − *β* ^2^ defines a critical surface in the parameter space.

We now consider the geometric phase of the nonlinear quantum system under discussion. In considering that our oscillator has a set of slow-varying parameters, we notice that as the rate of change of the parameters approaches zero, the ratio of the energy *E* to the frequency *ω* remains unchanged during the entire process so that the adiabatic condition is satisfied^37^. Hence, the action variable *I* = *E*/*ω* is an adiabatic invariant^38^ of the generalized harmonic oscillator. Therefore, when the Hamiltonian adiabatically evolves along a circuit *C* in parameter space after a long period *T*, the position of the oscillator on the ellipse is determined by an angle variable Θ conjugated to the action variable *I*, given by Θ = *ωT* + *γ*_*H*_, where *γ*_*H*_ is an additional shift in the angle variable caused by the cyclic adiabatic change of parameters. The relation between the pair of canonical coordinates and the action-angle variables can be specified by $\theta =\sqrt{\tfrac{2\gamma I}{\omega }}\,\cos \,{\rm{\Theta }}\,{\rm{and}}\,p=-\sqrt{\tfrac{2\gamma I}{\omega }}\,(\tfrac{\beta }{\gamma }\,\cos \,{\rm{\Theta }}+\tfrac{\omega }{\gamma }\,\sin \,{\rm{\Theta }})$. In terms of a differential form, the classical adiabatic angle, namely Hannay angle, is an integral of the angle 2-form^38^ ${\gamma }_{H}={\int }_{\partial S=C}W$, where *S* is an arbitrary open surface in the parameter space whose boundary is *C*. The angle 2-form for the generalized harmonic oscillator can be written explicitly as refs 6 and 7

5 $$W=\frac{\alpha d\beta \wedge d\gamma +\beta d\gamma \wedge d\alpha +\gamma d\alpha \wedge d\beta }{4{(\alpha \gamma -{\beta }^{2})}^{3/2}}.$$

To better display the geometry, we perform a change of variables *α* = *T* + *X*, *γ* = *T* − *X* and *β* = *Y*, and obtain

6 $$W=\frac{TdX\wedge dY+XdY\wedge dT+YdT\wedge dX}{2{({T}^{2}-{X}^{2}-{Y}^{2})}^{3/2}}.$$

If we denote *T*, *X* and *Y* as *X* ^0^, *X* ^1^ and *X* ^2^, the angle 2-form of the generalized harmonic oscillator is invariant under a local scale transformation *X*′^*μ*^ = *λ*(*X* ^*μ*^)*X* ^*μ*^ and an *SO*(2,1) transformation ${X}^{^{\prime} \mu }={{\rm{\Lambda }}}_{\nu }^{\mu }{X}^{\nu }$, where *λ*(*X* ^*μ*^) is a local scale factor and ${{\rm{\Lambda }}}_{\nu }^{\mu }$ is an element of the Lorentz group. An important feature is that when the oscillation frequency $\omega =\sqrt{{T}^{2}-{X}^{2}-{Y}^{2}}$ approaches zero, the angle 2-form exhibits a conic singularity in the parameter space (see Fig. 3a).

The conic singularity in parameter space is similar to the causal singularity of the light cone in Minkowski space. Such an analogue implies that the external parameters may be identified with the coordinates in Minkowski space with adequate constraints. A natural assumption is that spacetime is homogeneous and isotropic, such that we can define a cosmic clock by the oscillation frequency. We will demonstrate that a 2 + 1 dimensional de Sitter space is an adequate choice.

In the following, we will analyze the relationship between the classical adiabatic angle and the cosmology in 2 + 1 dimensions in detail. A 2 + 1 dimensional de Sitter space is defined as the set of all points (*T*, *X*, *Y*, *Z*) in a given 3 + 1 dimensional Minkowski space subjected to the constraint^34, 39^

7 $$-{T}^{2}+{X}^{2}+{Y}^{2}+{Z}^{2}={L}^{2},$$

where *L* is a parameter with units of length called the de Sitter radius, and the metric of the Minkowski space is given by

8 $$d{s}^{2}=-d{T}^{2}+d{X}^{2}+d{Y}^{2}+d{Z}^{2}.$$

De Sitter space is maximally symmetric^40^, and thus the Riemann curvature tensor for a de Sitter space is fully determined by the metric tensor, *R*_*μνλσ*_ = *g*_*μλ*_ *g*_*νσ*_ − *g*_*μσ*_ *g*_*νλ*_. Here, the Greek indices range over 0, 1 and 2. It is also straightforward to check that de Sitter space is a vacuum solution of Einstein’s field equation with a positive cosmological constant given by Λ = *L* ^−2^. In the following, we set *L* = 1. After contraction of indices, we obtain the Ricci tensor *R*_*μν*_ = 2*g*_*μν*_ and the scalar curvature *R* = 6 for a 2 + 1 dimensional de Sitter space. Among the various expressions of de Sitter space, the coordinate choice $Z=\,\cosh \,t$, $T=\,\sinh \,t\tilde{T}$, $X=\,\sinh \,t\tilde{X}$ and $Y=\,\sinh \,t\tilde{Y}$ yields $d{s}^{2}=-d{t}^{2}+{\sinh }^{2}\,td{\sigma }^{2}$, where $d{\sigma }^{2}=-d{\tilde{T}}^{2}+d{\tilde{X}}^{2}+d{\tilde{Y}}^{2}$ is the spatial metric with coordinates $\tilde{T}$, $\tilde{X}$ and $\tilde{Y}$ satisfying ${\tilde{T}}^{2}-{\tilde{X}}^{2}-{\tilde{Y}}^{2}=1$, which describes the two-dimensional unit hyperboloid. In the hyperbolic coordinates $\tilde{T}=\,\cosh \,\psi$, $\tilde{X}=\,\sinh \,\psi \,\cos \,\varphi$ and $\tilde{Y}=\,\sinh \,\psi \,\sin \,\varphi$, we obtain the standard metric on the unit hyperboloid, $d{\sigma }^{2}=d{\psi }^{2}+{\sinh }^{2}\,\psi d{\varphi }^{2}$, from which we see that the volume form of the unit hyperboloid is $\sinh \,\psi d\psi \wedge d\varphi$. Therefore, the metric of the 2 + 1 dimensional de Sitter space in the coordinates (*t*,*ψ*,*φ*) is precisely the Friedmann-Lemaître-Robertson-Walker metric^35^, which describes a homogeneous and isotropic expanding universe in 2 + 1 dimensions^34–36^

9 $$d{s}^{2}=-d{t}^{2}+{a}^{2}(t)\,(d{\psi }^{2}+{\sinh }^{2}\,\psi d{\varphi }^{2}),$$

where *t* ∈ (−∞, ∞) is the coordinate time, *ψ* ∈ (0, ∞) is the hyperbolic angle, *φ* ∈ (0, 2*π*) is the circular angle and $a(t)=\,\sinh \,t$ is the cosmic scale factor^35^ of the de Sitter universe. At a given time, the universe corresponds to a slice of the de Sitter hyperboloid at a fixed *Z*. At *t* = 0, spacetime degenerates into a single point (*T*, *X*, *Y*, *Z*) = (0, 0, 0, ±1), which corresponds to the big bang of the de Sitter universe^35^.

In the tetrad formalism of general relativity^28, 41, 42^, the vielbein, the connection form and the curvature form are the basic quantities. The vielbeins for the Friedmann-Lematre-Robertson-Walker metric are *e* ^0^ = *dt*, *e* ^1^ = *a*(*t*)*dψ* and ${e}^{2}=a(t)\,\sinh \,\psi d\varphi$. As the infinitesimal rotations of the vielbeins are described by the first Cartan structure equation^5^ $d{e}^{a}+{\omega }_{b}^{a}\wedge {e}^{b}=0$, the non-vanishing connection 1-forms are ${\omega }_{0}^{1}={\omega }_{1}^{0}=\dot{a}d\psi$, ${\omega }_{0}^{2}={\omega }_{2}^{0}=\dot{a}\,\sinh \,\psi d\varphi$ and ${\omega }_{1}^{2}=-{\omega }_{2}^{1}=\,\cosh \,\psi d\varphi$. The curvature 2-forms are obtained from the second Cartan structure equation ${R}_{b}^{a}=d{\omega }_{b}^{a}+{\omega }_{c}^{a}\wedge {\omega }_{b}^{c}$ and the non-vanishing curvature 2-forms are ${R}_{0}^{1}={R}_{1}^{0}=adt\wedge d\psi$, ${R}_{0}^{2}={R}_{2}^{0}=a\,\sinh \,\psi dt\wedge d\varphi$ and ${R}_{2}^{1}=-{R}_{1}^{2}={a}^{2}\,\sinh \,\psi d\psi \wedge d\varphi$.

After a straightforward computation, the curvature 2-form ${R}_{2}^{1}$ can be written in terms of the coordinates *T*, *X* and *Y* in the 3 + 1 dimensional Minkowski space as

10 $${R}_{2}^{1}={a}^{2}[\frac{TdX\wedge dY+XdY\wedge dT+YdT\wedge dX}{{({T}^{2}-{X}^{2}-{Y}^{2})}^{3/2}}].$$

Comparing Eqs (6) and (10), we immediately recognize that $W=\frac{1}{2{a}^{2}}{R}_{2}^{1}$, provided that the coordinates *T*, *X* and *Y* in the 3 + 1 dimensional Minkowski space are identified as the slow-varying parameters of the generalized harmonic oscillator. Under this key assumption, we obtain the relation *ω* ^2^ = *T* ^2^ − *X* ^2^ − *Y* ^2^ = *a* ^2^(*t*), which implies that the fundamental frequency of oscillation *ω* should be identified as the cosmic scale factor *a*(*t*) of the expanding universe. On the unit hyperboloid *T* ^2^ − *X* ^2^ − *Y* ^2^ = 1, the classical geometric phase of the generalized harmonic oscillator takes a simple form — the angle 2-form *W* is just half of the curvature 2-form ${R}_{2}^{1}$ of the de Sitter universe

11 $$W=\frac{1}{2}{R}_{2}^{1}.$$

After integration, the classical adiabatic angle *γ*_*H*_ around any circuit *C* in the parameter space is

12 $${\gamma }_{H}={\int }_{C}W=\frac{1}{2}{\int }_{0}^{2\pi }{\int }_{0}^{\psi (\varphi )}\sinh \,\psi d\psi d\varphi =\frac{1}{2}A(C),$$

where *A*(*C*) is the area on the unit hyperboloid subtended by *C* at the origin. As shown in Fig. 3, an arbitrary circuit *C* in the 2 + 1 dimensional de Sitter space can be projected onto the two-dimensional unit hyperboloid, and the resulting classical geometric phase is half of the solid angle subtended by the circuit at the origin. As the fundamental frequency of oscillation is now identified as the cosmic scale factor of the expanding universe, the conic singularity of the angle 2-form of the harmonic oscillator should be understood as the causal singularity of the de Sitter universe at the beginning of time (see Fig. 3), and the critical surface *T* ^2^ − *X* ^2^ − *Y* ^2^ = 0 in the parameter space is a single point (*T*, *X*, *Y*, *Z*) = (0, 0, 0, ±1) in the 2 + 1 dimensional de Sitter space where the proper distance vanishes.

## Discussion

### Bose-Einstein condensate as a realization of classical geometric phase

Here we outline an experimental proposal to realize classical geometric phase based on a Bose-Einstein condensate (BEC) in an asymmetric double-well potential. For fuller details, see SI, Note 1.

In this proposal, the BEC in a double-well potential is described by two weakly coupled macroscopic wave functions separated by a potential barrier^43^. Denoting the two wave amplitudes by *ψ*_1_ and *ψ*_2_, the Hamiltonian has the form

13 $$\begin{array}{rcl}H & = & {\epsilon }_{1}{|{\psi }_{1}|}^{2}+{\epsilon }_{2}{|{\psi }_{2}|}^{2}+\frac{{U}_{1}}{2}{|{\psi }_{1}|}^{4}+\frac{{U}_{2}}{2}{|{\psi }_{2}|}^{4}\\  &  & +(K+{U}_{12}{|{\psi }_{1}|}^{2}+{U}_{21}{|{\psi }_{2}|}^{2})\,({\psi }_{1}^{\ast }{\psi }_{2}+{\psi }_{1}{\psi }_{2}^{\ast })\\  &  & +2I{|{\psi }_{1}|}^{2}{|{\psi }_{2}|}^{2}+\frac{I}{2}({\psi }_{1}^{\ast 2}{\psi }_{2}^{2}+{\psi }_{1}^{2}{\psi }_{2}^{\ast 2}),\end{array}$$

where ${\epsilon }_{1}$ and ${\epsilon }_{2}$ are the single-mode energies, *U*_1_ and *U*_2_ are the on-site interaction energies, $K+{U}_{12}{|{\psi }_{1}|}^{2}+$ ${U}_{21}{|{\psi }_{2}|}^{2}$ is the renormalized tunneling rate that depends on the populations of the two condensates, and the last two terms proportional to *I* are the inter-well interaction and the inter-well pair tunneling respectively. The dynamics of the system is governed by only two variables: the fractional population imbalance *p* = (*N*_1_ − *N*_2_)/(*N*_1_ + *N*_2_) and the quantum relative phase *θ* = *θ*_2_ − *θ*_1_ between the left and right condensates. The resulting quantum dynamics in an asymmetric double-well potential is described by

14 $$\dot{p}={E}_{J}\sqrt{1-{p}^{2}}\,\sin \,\theta ,\,\dot{\theta }={E}_{C}-{E}_{J}\frac{p\,\cos \,\theta }{\sqrt{1-{p}^{2}}},$$

where ${E}_{J}={\rm{\Delta }}+\beta p+\alpha \sqrt{1-{p}^{2}}\,\cos \,\theta$ is the effective Josephson tunneling energy and ${E}_{C}=\epsilon +\gamma p+$ $\beta \sqrt{1-{p}^{2}}\,\cos \,\theta$ is the effective energy difference between the two condensates. Here the coefficients Δ, $\epsilon$, *α*, *β* and *γ* are given by Δ = 2*K* + *U*_12_ + *U*_21_, $\epsilon ={\epsilon }_{1}-{\epsilon }_{2}+({U}_{1}-{U}_{2})/2$, *α* = 2*I*, *β* = *U*_12_ − *U*_21_ and *γ* = (*U*_1_ + *U*_2_)/2 − *I*, where Δ is the static tunneling energy, $\epsilon$ is the difference between the single-mode energies, and where *α*, *β* and *γ* are determined by the overlap of the spatial wave functions that are localized in each well. The Josephson tunneling energy *E*_*J*_, which includes the nonlinear interaction effects explicitly, depends significantly on the values of *α* and *β*, which in turn depend on the inter-well pair tunneling rate and the difference between the interaction-assisted tunneling energies.

As expected from the Josephson effect, the population imbalance and the relative phase undergo harmonic oscillations surrounding the fixed points of the dynamics. The system has fixed points for *E*_*J*_ = *E*_*C*_ = 0, which are solved by

15 $$\bar{p}=\frac{\beta {\rm{\Delta }}-\alpha \epsilon }{\alpha \gamma -{\beta }^{2}},\,\sqrt{1-{\bar{p}}^{2}}\,\cos \,\bar{\theta }=\frac{\beta \epsilon -\gamma {\rm{\Delta }}}{\alpha \gamma -{\beta }^{2}}.$$

Specifically, for $\epsilon ={\rm{\Delta }}=0$, the mean values of the population imbalance and the relative phase are $\bar{p}=0$ and $\bar{\theta }=\pm \pi /2$, where the harmonic oscillations around the mean values are governed by $\dot{p}=\pm \beta p-\alpha \theta$ and $\dot{\theta }=\gamma p\mp \beta \theta$, which can be derived from the Hamiltonian of a generalized harmonic oscillator $H=(\alpha {\theta }^{2}\mp 2\beta p\theta +\gamma {p}^{2})/2$. This implies that an initial population imbalance and a small derivation from the *π*/2 phase induces a sinusoidal Josephson oscillation with a finite oscillation frequency $\omega =\sqrt{\alpha \gamma -{\beta }^{2}}$. In contrast, for a symmetric double-well potential, the Josephson oscillation is governed by $\dot{p}=({\rm{\Delta }}+\alpha )\theta$ and $\dot{\theta }=-({\rm{\Delta }}+\alpha -\gamma )p$, which only describes an ordinary harmonic oscillator, as $\dot{p}$ is linearly proportional to *θ* but not a linear combination of *p* and *θ*.

In a realistic experiment, the BEC after initial evaporative cooling is loaded into an optical effective double well trap, which is created by the superposition of a periodic potential with a harmonic trapping potential^32, 44^

16 $$V=\frac{m}{2}({\omega }_{x}^{2}{(x-{\rm{\Delta }}x)}^{2}+{\omega }_{y}^{2}{y}^{2}+{\omega }_{z}^{2}{z}^{2})+{V}_{0}\,{\cos }^{2}\,(\frac{\pi x}{d}),$$

where *ω*_*x*_, *ω*_*y*_ and *ω*_*z*_ are the harmonic trapping frequencies, *d* is the periodicity, *V*_0_ is the potential depth, and Δ*x* is the relative position shift between the two potentials. The initial population imbalance between the two wells is obtained by loading the condensate into an asymmetric double-well potential, which is created by a nonzero shift of the harmonic confinement with respect to the periodic potential. The parameters *α*, *β* and *γ* can be tuned slowly by adjusting the potential barrier, the harmonic trapping frequencies, and the relative shift between the two potentials independently^32, 45^.

### Magnetic system as a realization of classical geometric phase

We can formulate the coupled mode equations for a double-well BEC into nonlinear dynamics of a classical spin **S** as

17a $${\dot{S}}_{x}=-(\epsilon +\beta {S}_{x}+\gamma {S}_{z})\,{S}_{y},$$

17b $${\dot{S}}_{y}=(\epsilon +\beta {S}_{x}+\gamma {S}_{z})\,{S}_{x}-({\rm{\Delta }}+\alpha {S}_{x}+\beta {S}_{z})\,{S}_{z},$$

17c $${\dot{S}}_{z}=({\rm{\Delta }}+\alpha {S}_{x}+\beta {S}_{z})\,{S}_{y},$$

using the mapping ${S}_{x}={\psi }_{1}^{\ast }{\psi }_{2}+{\psi }_{1}{\psi }_{2}^{\ast }$, ${S}_{y}=-i({\psi }_{1}^{\ast }{\psi }_{2}-{\psi }_{1}{\psi }_{2}^{\ast })$ and *S*_*z*_ = |*ψ*_1_|^2^ − |*ψ*_2_|^2^. The spin components satisfy the standard Poisson bracket $\{{S}_{i},{S}_{j}\}={\epsilon }_{ijk}{S}_{k}$, and ${\rm{\Delta }}^{\prime} ={\rm{\Delta }}+\alpha {S}_{x}+\beta {S}_{z}$ and $\epsilon ^{\prime} =\epsilon +\beta {S}_{x}+\gamma {S}_{z}$ can be regarded as the effective magnetic fields in the *x* and *z* directions respectively. Eq. (17) describe the anisotropic interactions of a single spin in an effective external magnetic field, which can be derived from the Hamiltonian

18 $$H={\rm{\Delta }}{S}_{x}+\epsilon {S}_{z}+\frac{\alpha }{2}{S}_{x}^{2}+\beta {S}_{x}{S}_{z}+\frac{\gamma }{2}{S}_{z}^{2}.$$

Here, the spin has a magnetic easy axis along the *y*-axis; Δ and $\epsilon$ represent the transverse magnetic fields; *α*, *β* and *γ* are the second-order magnetic anisotropy parameters. If we write *α* = 2(*D* + *F*) and *γ* = 2(*D* − *F*), the spin Hamiltonian in zero field can be written as $H=-D{S}_{y}^{2}+F({S}_{x}^{2}-{S}_{z}^{2})+\beta {S}_{x}{S}_{z}$, where *D* represents the uniaxial anisotropy parameter and *F* represents the transverse anisotropy parameter^14^. For ${\bar{S}}_{y}=0$, the steady-state solutions for Eq. (17) are determined by

$$(\epsilon +\beta {\bar{S}}_{x}+\gamma {\bar{S}}_{z})\,{\bar{S}}_{x}-({\rm{\Delta }}+\alpha {\bar{S}}_{x}+\beta {\bar{S}}_{z})\,{\bar{S}}_{z}=0.$$

For ${\bar{S}}_{y}\ne 0$, the steady-state solutions for Eq. (17) are determined by $\epsilon +\beta {\bar{S}}_{x}+\gamma {\bar{S}}_{z}={\rm{\Delta }}+\alpha {\bar{S}}_{x}+\beta {\bar{S}}_{z}=0$, which are solved by

19 $${\bar{S}}_{x}=\frac{\beta \epsilon -\gamma {\rm{\Delta }}}{\alpha \gamma -{\beta }^{2}},\,{\bar{S}}_{z}=\frac{\beta {\rm{\Delta }}-\alpha \epsilon }{\alpha \gamma -{\beta }^{2}},\,{\bar{S}}_{y}=\pm \sqrt{1-{\bar{S}}_{x}^{2}-{\bar{S}}_{z}^{2}}.$$

**Figure 4:**
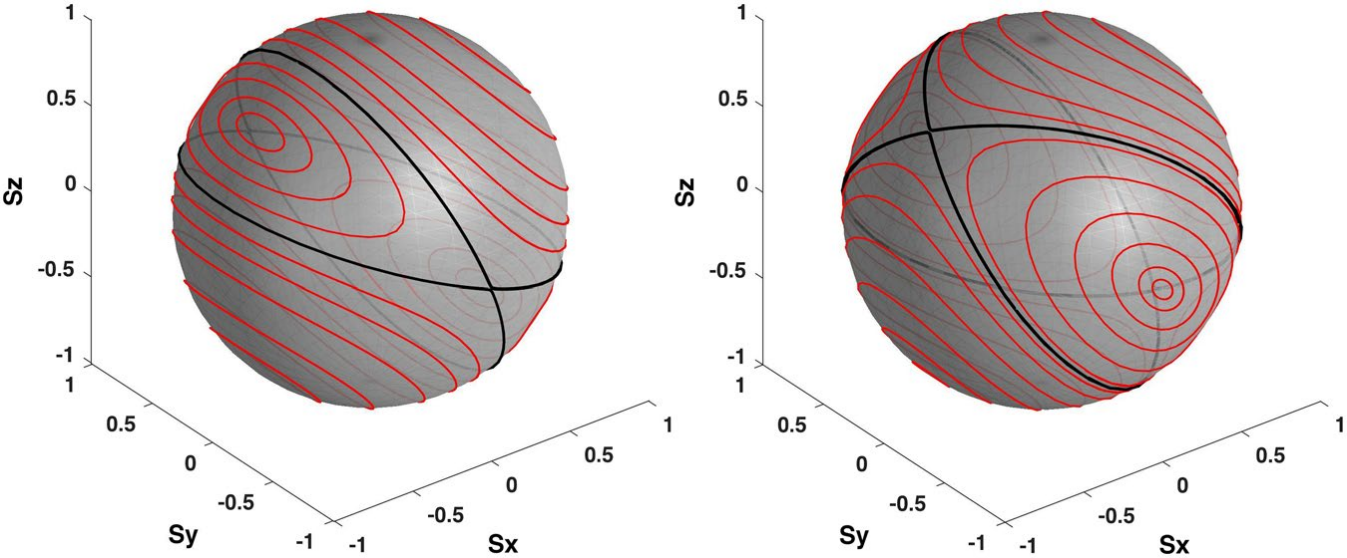
Classical spin representation of fixed points and Bogoliubov excitations of a double-well condensate for the symmetric case. The red curves show the phase trajectories for different energies $E=(\alpha {S}_{x}^{2}+2\beta {S}_{x}{S}_{z}+\gamma {S}_{z}^{2})/2$. The bold black lines indicate the separatrices which divide the sphere into four distinct regions. The left and right panels have the same values of *α* and *γ* (*α* = *γ* = 1) and the six fixed points are located at $({\bar{S}}_{x},{\bar{S}}_{y},{\bar{S}}_{z})=(0,\pm 1,0)$ and $(\pm 1/\sqrt{2},0,\pm 1/\sqrt{2})$ respectively. The left panel (*β* = 0.5) shows the phase diagram for *αγ* − *β* ^2^ > 0 in which the fixed points $({\bar{S}}_{x},{\bar{S}}_{y},{\bar{S}}_{z})=(0,\pm 1,0)$ are stable and the right panel (*β* = 1.5) shows the phase diagram for *αγ* − *β* ^2^ < 0 in which the points $({\bar{S}}_{x},{\bar{S}}_{y},{\bar{S}}_{z})=(0,\pm 1,0)$ become unstable.

In particular, for the case of zero field $(\epsilon ={\rm{\Delta }}=0)$, the solution $({\bar{S}}_{x},{\bar{S}}_{y},{\bar{S}}_{z})=(0,\pm 1,0)$ corresponds to a spin lying along the easy axis, namely the *y*-axis. This implies that the populations of the two condensates are the same and the two condensates have a *π*/2 phase difference. As shown in Fig. 4, the stability of the fixed points varies with the parameters $\epsilon$, Δ, *α*, *β* and *γ*. Therefore, it is also possible to use a classical magnet with nonlinear interactions^46, 47^ to simulate a nonlinear quantum system and the anomaly in gauge structures associated with critical surfaces.

### The classical adiabatic angle for a cycle near the critical surface

As seen from Eq. (6), the synthetic gauge field diverges at the critical surface *T* ^2^ − *X* ^2^ − *Y* ^2^ = 0. Here we show that the classical adiabatic angle accumulated along a cycle *C* near the critical surface in the parameter space also diverges. Let us denote *X* and *Y* as $R\,\cos \,\varphi$ and $R\,\sin \,\varphi$. Then the synthetic gauge field has the form

20 $$W=\frac{TRdR\wedge d\varphi -{R}^{2}dT\wedge d\varphi }{2{({T}^{2}-{R}^{2})}^{3/2}}.$$

The classical adiabatic angle *γ*_*H*_ can be evaluated as an integral of an open surface *S* whose boundary is *C*. After an integration over *φ* from 0 to 2*π*, we obtain

21 $${\gamma }_{H}(\eta )=\pi {\int }_{0}^{\eta }\frac{xdx}{{(1-{x}^{2})}^{3/2}}=\pi (\frac{1}{\sqrt{1-{\eta }^{2}}}-1),$$

where *x* ≡ *R*/*T* and 0 < *η* < 1 is a positive integer. Clearly, the classical adiabatic angle depends only on the ratio *R*/*T*. When the cycle is infinitesimally close to the critical surface *R* = *T*, the upper limit of the integral is equal to 1, and the resulting classical adiabatic angle $\mathop{\mathrm{lim}}\limits_{\eta \to 1}{\gamma }_{H}(\eta )=\pi \,\mathop{\mathrm{lim}}\limits_{\eta \to 1}\,{(1-{\eta }^{2})}^{-1/2}-\pi$ diverges. In this regard, the classical adiabatic angle is different from the Berry phase in the sense that both *γ*_*H*_ and *W* diverge at the critical surface, but the Berry phase may not necessarily diverge even when the gauge field does at the critical point.

### Prospects for further generalizations to higher dimensions

In this work, we study the synthetic gauge field associated with the classical adiabatic angle arising from adiabatic changes of a time-dependent Hamiltonian, and we found that the parameters of the Hamiltonian form a synthetic spacetime, that is, a 2 + 1 dimensional de Sitter space. The same reasoning may be applied to a Hamiltonian with more than three parameters. In such a case, the critical surface is no longer a quadratic cone, but is an algebraic surface with simple singularities. Therefore, a first step toward generalizations to higher dimensions requires a classification of simple singularities of algebraic surfaces. However, the resulting spacetime may not in general be a solution of Einstein’s field equation. Further investigation is needed to explore the relationship between the synthetic gauge field and the emergent spacetime for a Hamiltonian with more than three parameters.

## Electronic supplementary material

SUPPLEMENTARY INFORMATION for 2+1 dimensional de Sitter universe emerging from gauge structure of a nonlinear quantum system
