# Supplementary material for: 2 + 1 dimensional de Sitter universe emerging from the gauge structure of a nonlinear quantum system
Source: Sci Rep. 2017 Aug 29;7:9756. doi: 10.1038/s41598-017-08183-8 (PMC5574952; doi:10.1038/s41598-017-08183-8)
Supplement: Supplementary file 1 — SUPPLEMENTARY INFORMATION for 2+1 dimensional de Sitter universe emerging from gauge structure of a nonlinear quantum system [file 41598_2017_8183_MOESM1_ESM.pdf]

SUPPLEMENTARY INFORMATION for

2+1 dimensional de Sitter universe emerging as the gauge structure of a nonlinear quantum system

Chon-Fai Kam & Ren-Bao Liu

Note 1. Two-mode model for asymmetric double well BECs

The many-body Hamiltonian that describes interacting bosons confined by an external potential $V_{ext}(\mathbf{r})$ is

$$\hat{H}=\int d\mathbf{r}\hat{\Psi}^{\dagger}(\mathbf{r})\left[-\frac{\hbar^2\nabla^2}{2m}+V_{ext}(\mathbf{r})\right]\hat{\Psi}(\mathbf{r})+\frac{1}{2}\int d\mathbf{r}d\mathbf{r}'\hat{\Psi}^{\dagger}(\mathbf{r})\Psi^{\dagger}(\mathbf{r}')V(\mathbf{r}-\mathbf{r}')\hat{\Psi}(\mathbf{r}')\hat{\Psi}(\mathbf{r}), \quad \text{(A1)}$$

where $\hat{\Psi}(\mathbf{r})$ and $\hat{\Psi}^{\dagger}(\mathbf{r})$ are the boson field operators that annihilate and create a particle at $\mathbf{r}$ respectively and $V(\mathbf{r}-\mathbf{r}')$ is the two-body interatomic potential [S1]. In the Heisenberg representation for the field operators, the time evolution of the field operator is determined by the Heisenberg equation

$$i\frac{\partial\hat{\Psi}(\mathbf{r},t)}{\partial t}=[\hat{\Psi},\hat{H}]=\left[-\frac{\hbar^2\nabla^2}{2m}+V_{ext}(\mathbf{r})+\int d\mathbf{r}'\hat{\Psi}^{\dagger}(\mathbf{r}',t)V(\mathbf{r}-\mathbf{r}')\hat{\Psi}(\mathbf{r}',t)\right]\hat{\Psi}(\mathbf{r},t).$$

In a dilute ultracold atomic gas, only the elastic binary collisions between individual atoms are relevant. The binary collisions are characterized by a single s-wave scattering length a, which is irrelevant to the expressions of the two-body potential. Hence, we can replace the two-body potential $V(\mathbf{r}-\mathbf{r}')$ with an effective interaction $g\delta(\mathbf{r}-\mathbf{r}')$, which

results in $i\hbar\frac{\partial\hat{\Psi}(\mathbf{r},t)}{\partial t}=\left[-\frac{\hbar^2\nabla^2}{2m}+V_{ext}(\mathbf{r})+g\hat{\Psi}^\dagger(\mathbf{r},t)\hat{\Psi}(\mathbf{r},t)\right]\hat{\Psi}(\mathbf{r},t)$, where the coupling constant g is related to the scattering length through $g=4\pi\hbar^2 a/m$. When BEC occurs, we can replace the field operator $\hat{\Psi}(\mathbf{r},t)$ with its mean-field value $\Phi(\mathbf{r},t)\equiv\langle\hat{\Psi}(\mathbf{r})\rangle$ and obtain the time-dependent Gross-Pitaevskii equation for the condensate wave function [S2]

$$i\hbar\frac{\partial\Phi(\mathbf{r},t)}{\partial t}=\left(-\frac{\hbar^2\nabla^2}{2m}+V_{ext}(\mathbf{r})+g\,|\Phi(\mathbf{r},t)|^2\right)\Phi(\mathbf{r},t). \qquad \text{(A2)}$$

Here $\int|\Phi(\mathbf{r},t)|^2\,d\mathbf{r}=N$ is the number of condensed atoms. In a double-well potential, the Bose-Einstein condensate wave function $\Phi(\mathbf{r},t)$ can be written as a superposition of two time-independent spatial wave functions $\phi_1(\mathbf{r})$ and $\phi_2(\mathbf{r})$ that are localized in each well

$$\Phi(\mathbf{r},t)=\sqrt{N}[\psi_1(t)\phi_1(\mathbf{r})+\psi_2(t)\phi_2(\mathbf{r})], \qquad \text{(A3)}$$

where $\psi_1(t)$ and $\psi_2(t)$ are the time-dependent modal amplitudes. The condensate wave functions in the two wells $\phi_1(\mathbf{r})$ and $\phi_2(\mathbf{r})$ are assumed to be real valued functions satisfying the orthonormal condition

$$\int\phi_i(\mathbf{r})\phi_j(\mathbf{r})d\mathbf{r}=\delta_{ij}. \qquad \text{(A4)}$$

Hence $|\psi_1|^2$ and $|\psi_2|^2$ represent the occupation probabilities for the two modes, and the normalization condition for the condensate wave function $\int|\Phi(\mathbf{r},t)|^2\,d\mathbf{r}=N$ leads to the conservation of the occupation probabilities, $|\psi_1|^2+|\psi_2|^2=1$. Substitution of Eqs.

(A3) and (A4) into Eq. (A2) immediately yields

$$i\dot{\psi}_1 = \epsilon_1\psi_1 + K\psi_2 + U_1|\psi_1|^2\psi_1 + U_{12}(\psi_1^2\psi_2^* + 2|\psi_1|^2\psi_2)$$
$$+I(2\psi_1|\psi_2|^2 + \psi_1^*\psi_2^2) + U_{21}|\psi_2|^2\psi_2, \qquad \text{(A5a)}$$

$$i\dot{\psi}_2 = K\psi_1 + \epsilon_2\psi_2 + U_{12}|\psi_1|^2\psi_1 + I(\psi_1^2\psi_2^* + 2|\psi_1|^2\psi_2)$$
$$+U_{21}(2\psi_1|\psi_2|^2 + \psi_1^*\psi_2^2) + U_2|\psi_2|^2\psi_2, \qquad \text{(A5b)}$$

where the parameters ϵ_i, K, U_i, U_{ij} and I are given by the following overlap integrals

$$\epsilon_i = \int \phi_i(\mathbf{r})\left(-\frac{\hbar^2\nabla^2}{2m} + V_{ext}(\mathbf{r})\right)\phi_i(\mathbf{r})d\mathbf{r}, \qquad \text{(A6a)}$$

$$K = \int \phi_1(\mathbf{r})\left(-\frac{\hbar^2\nabla^2}{2m} + V_{ext}(\mathbf{r})\right)\phi_2(\mathbf{r})d\mathbf{r}, \qquad \text{(A6b)}$$

$$U_i = gN\int \phi_i^4(\mathbf{r})d\mathbf{r}, \qquad \text{(A6c)}$$

$$U_{ij} = gN\int \phi_i^3(\mathbf{r})\phi_j(\mathbf{r})d\mathbf{r}, \qquad \text{(A6d)}$$

$$I = gN\int \phi_1^2(\mathbf{r})\phi_2^2(\mathbf{r})d\mathbf{r}. \qquad \text{(A6e)}$$

Here ϵ_i are the single-mode energies, K is the tunneling rate of atoms between the two wells, and U_i are the on-site interaction energies. These parameters are the same as those defined for the standard two-mode model [S3]. The remaining parameters U_{12}, U_{21} and I include all the mixed terms in the spatial wave functions, and thus they are present only when the spatial wave functions have small but non-zero density on the other side. They were first introduced by Ananikian and Bergeman to include a renormalized tunneling rate to provide better agreement with numerical simulations and experimental results [S4]. To show this, we rewrite Eqs. (A5a)-(A5b) according to the

canonical formalism as $i\dot{\psi}_k = \partial H / \partial \psi_k^*$, where the classical Hamiltonian is

$$H = \epsilon_1 |\psi_1|^2 + \epsilon_2 |\psi_2|^2 + \frac{U_1}{2} |\psi_1|^4 + \frac{U_2}{2} |\psi_2|^4 + (K + U_{12} |\psi_1|^2 + U_{21} |\psi_2|^2)(\psi_1^* \psi_2 + \psi_1 \psi_2^*)$$
$$+ 2I |\psi_1|^2 |\psi_2|^2 + \frac{I}{2} (\psi_1^{*2} \psi_2^2 + \psi_1^2 \psi_2^{*2}). \qquad \text{(A7)}$$

The physical meaning of the parameters U_{12}, U_{21} and I can be understood from the Hamiltonian, where the $U_{12} |\psi_1|^2$ term contributes an interaction-assisted tunneling to the Hamiltonian and similarly for the $U_{21} |\psi_2|^2$ term. The last two terms in the Hamiltonian have different origins — the first term $2I |\psi_1|^2 |\psi_2|^2$ represents the inter-well interaction and the second term $\frac{I}{2} (\psi_1^{*2} \psi_2^2 + \psi_1^2 \psi_2^{*2})$ is the pair tunneling energy.

Quantitative analysis of the population variations of the two condensates and the macroscopic tunneling effects can be performed using the canonical formalism. Let us make the substitution $\psi_k = \sqrt{p_k} e^{i\theta_k}$, where p_1 and p_2 are the fractional populations of the Bose atoms at the two wells and θ_1 and θ_2 are the phases on the two sides of the barrier. If we define $\theta = \theta_2 - \theta_1$, Eqs. (A5a)-(A5b) can be written as

$$\dot{p}_1 = +2K' \sqrt{p_1 p_2} \sin\theta + 2I p_1 p_2 \sin 2\theta, \qquad \text{(A8a)}$$

$$\dot{p}_2 = -2K' \sqrt{p_1 p_2} \sin\theta - 2I p_1 p_2 \sin 2\theta, \qquad \text{(A8b)}$$

$$\dot{\theta}_1 = -\epsilon_1' - K_1' \sqrt{\frac{p_2}{p_1}} \cos\theta - 2I p_2 \cos^2\theta, \qquad \text{(A8c)}$$

$$\dot{\theta}_2 = -\epsilon_2' - K_2' \sqrt{\frac{p_1}{p_2}} \cos\theta - 2I p_1 \cos^2\theta. \qquad \text{(A8d)}$$

where $\epsilon_1' = \epsilon_1 + U_1 p_1 + I p_2$, $\epsilon_2' = \epsilon_2 + U_2 p_2 + I p_1$ are the single-mode energies modified by the nonlinear interactions, $K' = K + U_{12} p_1 + U_{21} p_2$ is the tunneling energy modified by the overlap of the spatial wave functions, $K_1' = K' + 2U_{12} p_1$ and $K_2' = K' + 2U_{21} p_2$. The first pair of equations implies $\dot{p}_1 = -\dot{p}_2$, which comes from the conservation of total population, $p_1 + p_2 = 1$. The atomic current cross the barrier is $N\dot{p}_1$, or $-N\dot{p}_2$. When p_1 and p_2 are approximately the same, the atomic current would be given by $J = J_0 \sin\theta + I_0 \sin 2\theta$, where $J_0 = NK'$ and $I_0 = NI/2$, and the phase evolution is determined by $\dot{\theta} = \epsilon_1 - \epsilon_2 + (K_1' - K_2')\cos\theta$. For the case when $I = 0$ and $U_{12} = U_{21}$, we recover the Josephson equations $J = J_0 \sin\theta$ and $\dot{\theta} = \epsilon_1 - \epsilon_2$ [S5]. If we define $p = p_1 - p_2$, the coupled-mode equations become

$$\dot{p} = (\Delta + \beta p)\sqrt{1-p^2}\sin\theta + I(1-p^2)\sin 2\theta, \qquad \text{(A9a)}$$

$$\dot{\theta} = \epsilon + \gamma p + \frac{\beta(1-2p^2) - \Delta p}{\sqrt{1-p^2}}\cos\theta - \alpha p \cos^2\theta, \qquad \text{(A9b)}$$

which can be derived from the Hamiltonian [S6, S7]

$$H = \epsilon p + \frac{\gamma}{2}p^2 + (\Delta + \beta p)\sqrt{1-p^2}\cos\theta + \frac{\alpha}{2}(1-p^2)\cos^2\theta.$$

Here the coefficients Δ, ϵ, α, β and γ are given by $\Delta = 2K + U_{12} + U_{21}$, $\epsilon = \epsilon_1 - \epsilon_2 + (U_1 - U_2)/2$, $\alpha = 2I$, $\beta = U_{12} - U_{21}$ and $\gamma = (U_1 + U_2)/2 - I$. Note that the nonlinear interactions produce a temporal change in the tunneling energy, and the tunneling energy $\Delta + \beta p$ is proportional to the population imbalance.

In a symmetric double well, we expect $\epsilon_1 = \epsilon_2$, $U_1 = U_2$ and $U_{12} = U_{21}$, which

implies $\epsilon = \beta = 0$. Then the time evolution for p and θ is governed by

$$\dot{p} = \Delta\sqrt{1-p^2}\sin\theta + I(1-p^2)\sin 2\theta, \tag{A10a}$$

$$\dot{\theta} = \gamma p + \frac{\beta(1-2p^2)-\Delta p}{\sqrt{1-p^2}}\cos\theta - \alpha p\cos^2\theta. \tag{A10b}$$

For $\overline{\theta} = 0$ or π, the fixed points of Eqs. (A10a)-(A10b) are $\overline{p} = 0$ and $\overline{p} = \pm\sqrt{1-\Lambda^2}$, where $\Lambda = \Delta/(\gamma-\alpha)$. By contrast, for the case when $\overline{\theta} \neq 0$ or π, the fixed points of Eqs. (A10a)-(A10b) are given by $\overline{p} = 0$ and $\cos\overline{\theta} = -\Delta/\alpha$. The oscillations of an initial population imbalance and phase difference are described by $\dot{p} = (\Delta+\alpha)\theta$ and $\dot{\theta} = -(\Delta+\alpha-\gamma)p$, which result in a finite oscillation frequency $\omega = \sqrt{(\Delta+\alpha)(\Delta+\alpha-\gamma)}$. In the absence of the bare tunneling parameter Δ, the frequency becomes $\sqrt{\alpha(\alpha-\gamma)}$.

We now discuss the time evolution of the condensates in an asymmetric double well. For $\sin\overline{\theta} = 0$, the fixed points of Eqs. (A9a)-(A9b) are solved by

$$\epsilon + (\gamma-\alpha)\overline{p} \pm \frac{\beta(1-2\overline{p}^2)-\Delta\overline{p}}{\sqrt{1-\overline{p}^2}} = 0$$

where the plus and minus signs correspond to $\overline{\theta} = 0$ and π respectively. For $\sin\overline{\theta} \neq 0$, the fixed points of the dynamics are determined by

$$\Delta + \beta\overline{p} + \alpha\sqrt{1-\overline{p}^2}\cos\overline{\theta} = \epsilon + \gamma\overline{p} + \beta\sqrt{1-\overline{p}^2} = 0, \tag{A11}$$

which has the solution $\overline{p} = \frac{\beta\Delta - \alpha\epsilon}{\alpha\gamma - \beta^2}$ and $\sqrt{1-\overline{p}^2}\cos\overline{\theta} = \frac{\beta\epsilon - \gamma\Delta}{\alpha\gamma - \beta^2}$. Specifically, for the

case where $\epsilon = \Delta = 0$, the fixed points are located at $(\bar{p},\bar{\theta}) = (0,\pm\pi/2)$, which implies a vanishing population imbalance and a $\pi/2$ phase difference. Near the fixed points $(\bar{p},\bar{\theta}) = (0,\pm\pi/2)$, the time evolution of the population imbalance and the relative phase is determined by $\dot{p} = \pm\beta p - \alpha\theta$ and $\dot{\theta} = \gamma p \mp \beta\theta$. Evidently, the atomic current is proportional to a linear combination of the population imbalance and the relative phase, and the relative phase is proportional to a linear combination of the population imbalance and the relative phase itself. For given values of α, β and γ, the time evolution of p and θ are sinusoidal, which can be easily observed from $\ddot{p} = -(\alpha\gamma - \beta^2)p$, $\ddot{\theta} = -(\alpha\gamma - \beta^2)\theta$, where $\omega = \sqrt{\alpha\gamma - \beta^2}$ is the frequency of oscillation. Hence, the time evolution of the population imbalance and the relative phase are governed by a generalized harmonic oscillator, where the Hamiltonian is defined by $H = (\alpha\theta^2 \mp 2\beta p\theta + \gamma p^2)/2$.

Note 2. Derivation of the Hannay angle

Here we consider an integrable classical system described by a Hamiltonian $H(p,q;\lambda(t))$, where $p = \{p_i\}$ and $q = \{q_j\}$ $(i, j \le N)$ are the canonical conjugate coordinates, and $\lambda = \{\lambda_\mu\}$ represent the slowly varying parameters. The classical adiabatic theorem [S11] ensures that if the system is initially located at a torus in the N-dimensional phase space with action $I = \{I_i\}$, it will remain on the torus with the

same values of *I*. The evolution of the conjugate angle coordinates $\theta = \{\theta_j\}$ is determined by the generating function $S^{(\alpha)}(q,I;\lambda(t))$ according to

$$\theta^{(\alpha)} = \frac{\partial S^{(\alpha)}}{\partial I}, \tag{B1a}$$

$$S^{(\alpha)}(q,I;\lambda(t)) \equiv \int_0^q p^{(\alpha)}(q,I;\lambda(t))dq, \tag{B1b}$$

where the superscript α labels the branches where p is a single-valued function of q. After the canonical transformation from $\{p,q\}$ to $\{I,\theta\}$, the new Hamiltonian $H'(\theta,I,t)$ differs from the angle-independent Hamiltonian $H(I;\lambda(t))$ by an amount proportional to the rate of change of λ

$$H'(\theta,I,t) = H(I;\lambda(t)) + \frac{d\lambda}{dt}\frac{\partial}{\partial\lambda}S^{(\alpha)}(q,I;\lambda(t)). \tag{B2}$$

As q can be regarded as a single-valued function of I and θ, we define a new single-valued function $F(\theta,I;\lambda)$ as

$$F(\theta,I;\lambda) \equiv S^{(\alpha)}(q(\theta,I;\lambda),I;\lambda(t)). \tag{B3}$$

From the definition of $F(\theta,I;\lambda)$, we have

$$\frac{\partial S^{(\alpha)}}{\partial\lambda} = \frac{\partial F}{\partial\lambda} - \frac{\partial S^{(\alpha)}}{\partial q}\frac{\partial q}{\partial\lambda} = \frac{\partial F}{\partial\lambda} - p^{(\alpha)}\frac{\partial q}{\partial\lambda}. \tag{B4}$$

Substituting Eq. (B4) into Eq. (B2), the Hamiltonian $H'(\theta,I,t)$ becomes

$$H'(\theta,I,t)=H(I;\lambda(t))+\frac{d\lambda}{dt}\left(\frac{\partial F}{\partial\lambda}(\theta,I;\lambda(t))-p(\theta,I;\lambda(t))\frac{\partial q}{\partial\lambda}(\theta,I;\lambda(t))\right). \quad \text{(B5)}$$

As p and q are now both single-valued functions of I and θ, we can remove the superscript α of $p(\theta,I;\lambda(t))$. Now, using the Hamilton equations for the angle variables $d\theta/dt=\partial H'/\partial I$ and integrating from 0 to T, we obtain [S12]

$$\theta(T)=\theta(0)+\int_0^T dt\omega(I;\lambda)+\int_0^T dt\frac{d\lambda}{dt}\frac{\partial}{\partial I}\left(\frac{\partial F}{\partial\lambda}-p\frac{\partial q}{\partial\lambda}\right),$$

where $\omega\equiv\{\omega_j\}$ with $\omega_j(I;\lambda)\equiv\partial H(I;\lambda)/\partial I_j$ are the instantaneous frequencies for fixed parameters, and the third term as a whole are the shifts in the angles $\theta=\{\theta_j\}$ in response to the adiabatic changes of parameters. To simplify the integration, we average out the fast oscillations by integrating over the torus

$$\Delta\theta(I;C)=\int_C d\lambda\frac{\partial}{\partial I}\frac{1}{(2\pi)^N}\prod_{j=1}^{N}\int_0^{2\pi}d\theta_j\left(\frac{\partial F}{\partial\lambda}-p\frac{\partial q}{\partial\lambda}\right), \quad \text{(B6)}$$

where the integration is performed over a closed curve C in the parameter space. The first term inside the parenthesis $\partial F/\partial\lambda$ is just a gradient and vanishes identically after integration. Therefore, the shifts in the angle variables can be transformed into an integral over a surface A whose boundary is C in the parameter space

$$\Delta\theta(I;C)=\int_{\partial A=C}W(I;\lambda), \quad \text{(B7)}$$

where W is the angle 2-form determined by

$$W(I;\lambda) = -\frac{\partial}{\partial I}\frac{1}{(2\pi)^N}\prod_{j=1}^{N}\int_0^{2\pi} d\theta_j dp \wedge dq. \tag{B8}$$

A first example of the angle 2-form is the generalized harmonic oscillator whose Hamiltonian is described by

$$H = \frac{1}{2}[\alpha(t)q^2 + 2\beta(t)pq + \gamma(t)p^2] \tag{B9}$$

where α, β and γ are some time-dependent external parameters. The angle-independent Hamiltonian $H'(I;\lambda)$ has the form $H'(I;\lambda) = I\omega$, where $\omega = \sqrt{\alpha\gamma - \beta^2}$ is the frequency of oscillation, and the Hamilton equations for fixed parameters are solved by $p(\theta, I;\lambda)$ and $q(\theta, I;\lambda)$, where $p(\theta, I;\lambda)$ and $q(\theta, I;\lambda)$ are single-valued functions of the action-angle coordinates, which are determined by

$$q = \sqrt{\frac{2\gamma I}{\omega}}\cos\theta, \tag{B10a}$$

$$p = -\sqrt{\frac{2\gamma I}{\omega}}\left(\frac{\beta}{\gamma}\cos\theta + \frac{\omega}{\gamma}\sin\theta\right). \tag{B10b}$$

Substituting Eq. (B10a)-(B10b) into Eq. (B8), we immediately obtain

$$W = \frac{1}{\pi}\int_0^{2\pi} d\theta \cos^2\theta d\left(\frac{\beta}{\gamma}\sqrt{\frac{\gamma}{\omega}}\right) \wedge d\sqrt{\frac{\gamma}{\omega}} = \frac{1}{2}d\left(\frac{\beta}{\gamma}\right) \wedge d\left(\frac{\gamma}{\sqrt{\alpha\gamma - \beta^2}}\right). \tag{B11}$$

After a short calculation, we get the final expression of the angle 2-form for a generalized harmonic oscillator

$$W = \frac{\alpha d\beta \wedge d\gamma + \beta d\gamma \wedge d\alpha + \gamma d\alpha \wedge d\beta}{4(\alpha\gamma - \beta^2)^{3/2}}. \tag{B12}$$

Note 3. Derivation of the curvature 2-form for the de Sitter space

Here we consider the vielbein formalism of the connection and curvature in classical general relativity [S13, S14]. In the vielbein formalism, the metric tensor is expressed as

$$g_{\mu\nu} \equiv e_\mu^\alpha e_\nu^\beta \eta_{\alpha\beta}, \tag{C1}$$

where $\eta_{\alpha\beta}$ is the conventional Minkowski metric; μ, ν are world indices; and α, β are Lorentz indices. The fundamental quantities are the vielbeins e_μ^α, which form an $n \times n$ invertible matrix and satisfy the orthogonality conditions

$$e_\alpha^\mu e_\nu^\alpha = \delta_\nu^\mu, \tag{C2a}$$

$$e_\mu^\alpha e_b^\mu = \delta_\beta^\alpha. \tag{C2b}$$

An immediate application of this formalism is to express the connection and curvature in terms of the vielbeins, or equivalently the vielbein 1-form $e^\alpha = e_\mu^\alpha dx^\mu$. The connection and the curvature are then defined by

$$de^\alpha + \omega_\beta^\alpha \wedge e^\beta = 0, \tag{C3a}$$

$$d\omega_\beta^\alpha + \omega_\gamma^\alpha \wedge \omega_\beta^\gamma = R_\beta^\alpha. \tag{C3b}$$

where $\omega_\beta^\alpha = \omega_{\beta\mu}^\alpha dx^\mu$ is the connection 1-form which is anti-symmetric in the Lorentz

indices, $\omega_{\alpha\beta} = -\omega_{\beta\alpha}$, and $R^{\alpha}_{\beta} = \frac{1}{2} R^{\alpha}_{\beta\mu\nu} dx^{\mu} \wedge dx^{\nu}$ is the curvature 2-form. Suppressing the Lorentz indices, we get Cartan's first and second structure equations

$$de + \omega \wedge e = 0, \tag{C4a}$$

$$d\omega + \omega \wedge \omega = R. \tag{C4b}$$

It should be noted that, for a local Lorentz transformation $e = \Lambda \tilde{e}$, the connection and the curvature transform as

$$\omega = \Lambda \tilde{\omega} \Lambda^{-1} - d\Lambda \Lambda^{-1}, \tag{C5a}$$

$$R = \Lambda \tilde{R} \Lambda^{-1}. \tag{C5b}$$

where $d\Lambda = \partial_{\mu} \Lambda dx^{\mu}$ is the infinitesimal Lorentz transform. The connection coefficients $\omega^{\alpha}_{\beta\mu}$ and the ordinary Christoffel symbols $\Gamma^{\mu}_{\nu\lambda}$ are related by [S15]

$$\Gamma^{\mu}_{\nu\lambda} = e^{\mu}_{\alpha} \partial_{\nu} e^{\alpha}_{\lambda} + e^{\mu}_{\alpha} e^{\beta}_{\lambda} \omega^{\alpha}_{\beta\nu}, \tag{C6}$$

while the curvature coefficients $R^{\alpha}_{\beta\mu\nu}$ and the conventional Riemann curvature tensors $R^{\rho}_{\sigma\mu\nu}$ are related by [S15]

$$R^{\rho}_{\sigma\mu\nu} = e^{\rho}_{\alpha} e^{\beta}_{\sigma} R^{\alpha}_{\beta\mu\nu}. \tag{C7}$$

A first example of connection and curvature forms are those for the 2+1 dimensional de

Sitter space, which is defined by the set of all points (T, X, Y, Z) in the 3+1 dimensional Minkowski space subjected to the constraint

$$-T^2 + X^2 + Y^2 + Z^2 = 1, \tag{C8}$$

where the metric of the Minkowski space is given by

$$ds^2 = -dT^2 + dX^2 + dY^2 + dZ^2. \tag{C9}$$

For the coordinate choice $Z = \cosh t$, $T = \sinh t \cosh\psi$, $X = \sinh t \sinh\psi \cos\phi$ and $Y = \sinh t \sinh\psi \sin\phi$, the metric describes a homogeneous and isotropic open universe

$$ds^2 = -dt^2 + a^2(t)(d\psi^2 + \sinh^2\psi\, d\phi^2), \tag{C10}$$

where $a(t) = \sinh t$ is the cosmological scale factor. The homogeneous and isotropic two-dimensional surfaces at constant t provide a natural slicing of spacetime. In this slicing, the metric can be written as $ds^2 = -dt^2 + \gamma_{ij}(x,t)dx^i dx^j$, where $\gamma_{ij}(x,t)$ are the spatial components of the metric tensor, which are explicitly written as $\gamma_{11} = a^2$ and $\gamma_{22} = a^2 \sinh^2\psi$. In these coordinates, the components of the extrinsic curvature are simply given by $K_{ij} \equiv \frac{1}{2}\partial_t \gamma_{ij}$ and hence we have $K_{11} = a\dot{a}$ and $K_{22} = a\dot{a}\sinh^2\psi$. The mean curvature K is the trace of the extrinsic curvature K_{ij}

$$K \equiv \gamma^{ij} K_{ij} = \frac{1}{2}\partial_t \ln\gamma = \frac{2\dot{a}}{a}, \tag{C11}$$

where γ is the determinant of the spatial metric tensor γ_{ij}. As the mean curvature K is proportional to the Hubble parameter $H \equiv \dot{a}/a$, it can be regarded as a measure of spatial expansion of the expanding universe. The vielbein 1-forms can be obtained directly from the metric

$$e^0 = dt,\ e^1 = a(t)d\psi,\ e^2 = a(t)\sinh\psi d\phi. \tag{C12}$$

A straightforward computation yields

$$\omega_0^1 = \dot{a}d\psi,\ \omega_0^2 = \dot{a}\sinh\psi d\phi,\ \omega_1^2 = \cosh\psi d\phi, \tag{C13}$$

where the connection 1-forms satisfy $\omega_a^0 = \omega_0^a$ and $\omega_b^a = -\omega_a^b$. Here a and b denote spatial indices. Employing Cartan's second structure equation, we obtain

$$R_1^0 = a dt \wedge d\psi = e^0 \wedge e^1, \tag{C14a}$$

$$R_2^0 = a\sinh\psi dt \wedge d\phi = e^0 \wedge e^2, \tag{C14b}$$

$$R_2^1 = a^2 \sinh\psi d\psi \wedge d\phi = e^1 \wedge e^2. \tag{C14c}$$

Similarly, the curvature 2-forms satisfy $R_a^0 = R_0^a$ and $R_b^a = -R_a^b$. As the curvature 2-forms obey the simple relation $R_\beta^\alpha = e^\alpha \wedge e^\beta$, the Riemann curvature tensors in the vielbein formalism are given by

$$R^0_{101} = R^0_{202} = R^1_{212} = 1, \quad \text{(C15)}$$

which satisfy $R^0_{a0a} = -R^a_{0a0}$ and $R^a_{bab} = R^b_{aba}$. Thus the Ricci tensors in the vielbein formalism are

$$\begin{aligned} R_{00} &= R^1_{010} + R^2_{020} = -2, \\ R_{11} &= R^0_{101} + R^2_{121} = 2, \\ R_{22} &= R^1_{010} + R^2_{020} = 2. \end{aligned} \quad \text{(C16)}$$

The above shows that the Ricci tensors obey the simple relation $R_{\alpha\beta} = 2\eta_{\alpha\beta}$. As a result, the scalar curvature R becomes

$$R = -R_{00} + R_{11} + R_{22} = 6. \quad \text{(C17)}$$

We would now like to express the curvature 2-forms R^0_1, R^0_2 and R^1_2 in terms of T, X and Y. If we denote the coordinates of the unit hyperboloid satisfying $\tilde{T}^2 - \tilde{X}^2 - \tilde{Y}^2 = 1$ as $\tilde{T} \equiv \cosh\psi$, $\tilde{X} \equiv \sinh\psi\cos\phi$ and $\tilde{Y} \equiv \sinh\psi\sin\phi$, then a straightforward calculation gives

$$\begin{aligned} \tilde{T}d\tilde{X} \wedge d\tilde{Y} &= \cosh^2\psi \sinh\psi d\psi \wedge d\phi, \\ \tilde{X}d\tilde{Y} \wedge d\tilde{T} &= -\sinh^3\psi \cos^2\phi d\psi \wedge d\phi, \\ \tilde{Y}d\tilde{T} \wedge d\tilde{X} &= -\sinh^3\psi \sin^2\phi d\psi \wedge d\phi. \end{aligned}$$

These immediately lead to

$$\tilde{T}d\tilde{X} \wedge d\tilde{Y} + \tilde{X}d\tilde{Y} \wedge d\tilde{T} + \tilde{Y}d\tilde{T} \wedge d\tilde{X} = \sinh\psi d\psi \wedge d\phi, \quad \text{(C18)}$$

which is the area element of the unit hyperboloid. If t is now allowed to vary over $-\infty < t < \infty$, substitution of $(T, X, Y) = (a\tilde{T}, a\tilde{X}, a\tilde{Y})$ yields

$$
\begin{aligned}
TdX \wedge dY &= a^2(\tilde{T}\tilde{X}da \wedge d\tilde{Y} - \tilde{Y}\tilde{T}da \wedge d\tilde{X} + a\tilde{T}d\tilde{X} \wedge d\tilde{Y}), \\
XdY \wedge dT &= a^2(\tilde{X}\tilde{Y}da \wedge d\tilde{T} - \tilde{T}\tilde{X}da \wedge d\tilde{X} + a\tilde{X}d\tilde{Y} \wedge d\tilde{T}), \\
YdT \wedge dX &= a^2(\tilde{Y}\tilde{T}da \wedge d\tilde{X} - \tilde{X}\tilde{Y}da \wedge d\tilde{X} + a\tilde{Y}d\tilde{T} \wedge d\tilde{X}).
\end{aligned}
$$

After summation, we have the relation

$$
\begin{aligned}
&TdX \wedge dY + XdY \wedge dT + YdT \wedge dX \\
&= a^3(\tilde{T}d\tilde{X} \wedge d\tilde{Y} + \tilde{X}d\tilde{Y} \wedge d\tilde{T} + \tilde{Y}d\tilde{T} \wedge d\tilde{X}).
\end{aligned}
$$

Recognizing $\sqrt{T^2 - X^2 - Y^2}$ as the cosmological scale factor $a(t)$, we obtain the formula

$$\frac{TdX \wedge dY + XdY \wedge dT + YdT \wedge dX}{(T^2 - X^2 - Y^2)^{3/2}} = a^{-2}R_2^1. \tag{C19}$$

As a remark, for a closed curve $C = \partial S$ in the parameter space $\left(T, X, Y\right)$, the integration of the 2-form $a^{-2}R_2^1$ over S always gives the area enclosed by the projection of C on the unit hyperboloid. Now, a straightforward computation gives

$$\frac{dX \wedge dY}{\sqrt{X^2 + Y^2}} = d(a\sinh\psi) \wedge d\phi = a\left(\frac{d\tilde{X} \wedge d\tilde{Y}}{\sqrt{\tilde{X}^2 + \tilde{Y}^2}}\right) + \frac{\dot{a}}{a}R_2^0.$$

As a result, we obtain the simple formula

$$\frac{dX \wedge dY}{\sqrt{\gamma}} - \frac{d\tilde{X} \wedge d\tilde{Y}}{\sqrt{\tilde{\gamma}}} = \frac{H}{a}R_2^0, \tag{C20}$$

where $H \equiv \dot{a}/a$ is the Hubble parameter, γ is the determinant of the spatial metric tensor γ_{ij} and $\tilde{\gamma}$ is the determinant of the spatial metric tensor evaluated on the unit hyperboloid. Hence the 2-form R_2^0 is proportional to the difference between the renormalized area element in the horizontal XY-plane and its projection on the unit hyperboloid, with $\sqrt{\gamma}$ being the normalized factor. Similarly, we have the formula

$$dT \wedge dL = ada \wedge d\psi = aHR_1^0, \tag{C21}$$

where $L \equiv \sqrt{X^2 + Y^2} = a\sinh\psi$. Evidently, the 2-form R_1^0 is proportional to the area element in the vertical TL-plane.

As a final remark, the other two curvature 2-forms R_1^0 and R_2^0 are not directly related to the angle 2-form W, but they can still be expressed in terms of T, X and Y : $R_1^0 = (aH)^{-1} dT \wedge dL$ and $R_2^0 = aH^{-1}(\sqrt{|g|^{-1}} dX \wedge dY - \sqrt{|\tilde{g}|^{-1}} d\tilde{X} \wedge d\tilde{Y})$, where $L \equiv \sqrt{X^2 + Y^2}$, $H \equiv \dot{a}/a$ is the Hubble parameter and $g \equiv \det g_{\mu\nu}$ is the determinant of the metric tensor. Hence, the 2-form R_1^0 is proportional to the area element in the TL-plane and the 2-form R_2^0 is proportional to the difference between the renormalized area element in the XY-plane and its projection on the unit hyperboloid.

Note 4. Relationship between Bogoliubov excitation spectrum and the fundamental frequencies

Bogoliubov excitations are collective excitations that involve the motion of the whole condensate. For such a motion, the condensate wave function $\mathbf{\Phi}(\mathbf{r},t)$ can be written as a superposition of the time-independent condensate ground sate and the small amplitude harmonic perturbations on the condensate ground sate

$$\mathbf{\Phi}(\mathbf{r},t)=\mathbf{\Phi}(\mathbf{r})+u(\mathbf{r})e^{-i\omega t}+v(\mathbf{r})e^{i\omega t}, \tag{D1}$$

where $\mathbf{\Phi}(\mathbf{r})$ is the condensate ground state, $u(\mathbf{r})$ and $v(\mathbf{r})$ are the small amplitude oscillations, namely Bogoliubov excitations, on the condensate ground state, and ω is a frequency from the corresponding Bogoliubov excitation spectrum. In the following, we will show by explicit calculation that the Bogoliubov excitation spectrum is identical to the fundamental frequency of periodic orbits around the fixed points.

As we have discussed in Note. 1, the dynamics of the condensate in an asymmetric doubled-well potential is described by Eqs. (A9a)-(A9b). For the special case where $\epsilon=\Delta=0$, the Hamiltonian is written as

$$H=\frac{\gamma}{2}p^2+\beta p\sqrt{1-p^2}\cos\theta+\frac{\alpha}{2}(1-p^2)\cos^2\theta, \tag{D2}$$

where the fixed points for $\sin\bar{\theta}\neq 0$ are given by $(\bar{p},\bar{\theta})=(0,\pm\pi/2)$, which are equivalent to $\bar{p}_1=\bar{p}_2=1/2$ and $\bar{\theta}_2-\bar{\theta}_1=\pm\pi/2$ according to $\bar{p}\equiv\bar{p}_1-\bar{p}_2$ and $\bar{\theta}\equiv\bar{\theta}_2-\bar{\theta}_1$. From the definition of the wave amplitudes $\psi_k=\sqrt{p_k}e^{i\theta_k}$, we obatin

$$H=\frac{\gamma}{2}\left(|\psi_1|^2-|\psi_2|^2\right)^2+\beta\left(|\psi_1|^2-|\psi_2|^2\right)\left(\psi_1\psi_2^*+\psi_2\psi_1^*\right)+\frac{\alpha}{2}\left(\psi_1\psi_2^*+\psi_2\psi_1^*\right)^2. \tag{D3}$$

The Hamiltonian (D3) is a homogeneous function of the wave amplitudes ψ_1 and ψ_2

of degree 2, which satisfies $H(z\psi_1, z\psi_2) = z^2 H(\psi_1, \psi_2)$ for any complex number z. To separate the global phase $\lambda \equiv (\theta_2 + \theta_1)/2$, we write $(\psi_1, \psi_2) \equiv e^{i\lambda}(\sqrt{p_1} e^{-i\theta/2}, \sqrt{p_2} e^{i\theta/2})$. A direction calculation shows that

$$\dot{\lambda} = p\dot{q} - \sum_k \psi_k^* \frac{\partial H}{\partial \psi_k^*} = p\dot{q} - 2H, \tag{D4}$$

where the last step of Eq. (D4) comes from the fact that $H(\psi_1, \psi_2)$ is a homogeneous function of degree 2. Substituting $\bar{p}_1 = \bar{p}_2 = 1/2$ and $\bar{\theta} = \pm\pi/2$ into $\psi_k = \sqrt{p_k} e^{i\theta_k}$, we obtain $(\bar{\psi}_1, \bar{\psi}_2) = \frac{1}{2}(1 \mp i, 1 \pm i)$, where the global phase for the fixed points vanishes. Expanding $\psi_k = \sqrt{p_k} e^{i\theta_k}$ around the fixed points, we obtain $\psi_k = \bar{\psi}_k (1 + \delta p_k + \delta\theta_k)$, where δp_k and $\delta\theta_k$ are the harmonic oscillations around the fixed points. For fixed parameters, as a result of Eqs. (B10a)-(B10b), the harmonic oscillations surrounding the fixed points $(\bar{p}, \bar{\theta}) = (0, \pm\pi/2)$ can be expressed as

$$\delta\theta = \sqrt{\frac{2\gamma I}{\omega}} \cos\omega t, \tag{D5a}$$

$$\delta p = -\sqrt{\frac{2\gamma I}{\omega}} \left(\frac{\beta}{\gamma} \cos\omega t + \frac{\omega}{\gamma} \sin\omega t \right), \tag{D5b}$$

where I is the action variable, which is the area enclosed by a given periodic orbit, and $\omega \equiv \sqrt{\alpha\gamma - \beta^2}$ is the fundamental frequency around the fixed points. Substitution of Eqs. (D5a)-(D5b) into $\psi_k = \bar{\psi}_k (1 + \delta p_k + \delta\theta_k)$, we have

$$\psi_1 = \bar{\psi}_1 - \bar{\psi}_1 \sqrt{\frac{I}{8\omega\gamma}} \left[\left(\beta+\gamma+\omega\right) e^{i\omega t} + i\left(\beta+\gamma-\omega\right) e^{-i\omega t} \right], \tag{D6a}$$

$$\psi_2 = \bar{\psi}_2 + \bar{\psi}_2 \sqrt{\frac{I}{8\omega\gamma}} \left[\left(\beta+\gamma+\omega\right) e^{i\omega t} + i\left(\beta+\gamma-\omega\right) e^{-i\omega t} \right], \tag{D6b}$$

Substitution of Eqs. (D6a)-(D6b) into Eq. (A3) immediately yields

$$\begin{aligned}\Phi(\mathbf{r},t) = {} & \sqrt{N}[\bar{\psi}_1\phi_1(\mathbf{r}) + \bar{\psi}_2\phi_2(\mathbf{r})] \\ & -\bar{\psi}_1 \sqrt{\frac{NI}{8\omega\gamma}} \left[\left(\beta+\gamma+\omega\right) e^{i\omega t} + i\left(\beta+\gamma-\omega\right) e^{-i\omega t} \right] \phi_1(\mathbf{r}) \\ & +\bar{\psi}_2 \sqrt{\frac{NI}{8\omega\gamma}} \left[\left(\beta+\gamma+\omega\right) e^{i\omega t} + i\left(\beta+\gamma-\omega\right) e^{-i\omega t} \right] \phi_2(\mathbf{r}).\end{aligned} \tag{D7}$$

If we write $\Phi(\mathbf{r}) \equiv \sqrt{N}[\bar{\psi}_1\phi_1(\mathbf{r}) + \bar{\psi}_2\phi_2(\mathbf{r})]$ and

$$u(\mathbf{r}) = i\sqrt{\frac{NI}{8\omega\gamma}} \left(\beta+\gamma-\omega\right)\left(\bar{\psi}_2\phi_2(\mathbf{r}) - \bar{\psi}_1\phi_1(\mathbf{r})\right), \tag{D8a}$$

$$v(\mathbf{r}) = \sqrt{\frac{NI}{8\omega\gamma}} \left(\beta+\gamma+\omega\right)\left(\bar{\psi}_2\phi_2(\mathbf{r}) - \bar{\psi}_1\phi_1(\mathbf{r})\right), \tag{D8b}$$

Eq. (D7) reduces to Eq. (D1). The above shows explicitly that the Bogoliubov excitation spectrum of the corresponding eigenstates is identical to the fundamental frequencies of the periodic oscillations around the fixed points.

Supplementary References

S1. Dalfovo, F., Giorgini, S., Pitaevskii, L. P. & Stringari, S. Theory of Bose-Einstein condensation in trapped gases. *Rev. Mod. Phys.* **71**, 463 (1999).

S2. Leggett, A. J. Bose-Einstein condensation in the alkali gases: Some fundamental concepts. *Rev. Mod. Phys.* **73**, 307 (2001).

S3. Smerzi, A., Fantoni, S., Giovanazzi, S. & Shenoy, S. R. Quantum coherent atomic tunneling between two trapped Bose-Einstein condensates. *Phys. Rev. Lett.* **79**, 4950 (1997).

S4. Ananikian, D. & Bergeman, T. Gross-Pitaevskii equation for Bose particles in a double-well potential: Two-mode models and beyond. *Phys. Rev. A.* **73**, 013604 (2006).

S5. Levy, S., Lahoud, E., Shomroni, I. & Steinhauer, J. The ac and dc Josephson effects in a Bose–Einstein condensate. *Nature* **449**, 579 (2007).

S6. Barberis-Blostein, P. & Fuentes-Schuller, I. Mode-exchange collisions in an exactly solvable two-mode Bose-Einstein condensate. *Phys. Rev. A.* **78**, 013641 (2008).

S7. Cataldo, H. M. & Jezek, D. M. Dynamics in asymmetric double-well condensates. *Phys. Rev. A.* **90**, 043610 (2014).

S8. Arnold, V. I. *Mathematical methods of classical mechanics* (Springer, 1989).

S9. Berry, M. V. Classical adiabatic angles and quantal adiabatic phase. J. Phys. A: Math. Gen. **18**, 15-27 (1985).

S10. Wald, R. M. *General Relativity* (The University of Chicago Press, 1984).

S11. Carmeli, M. *Classical fields: General Relativity and Gauge Theory* (John Wiley & Sons, Inc., 1982).

S12. Carroll, S. M. *Spacetime and geometry: An introduction to general relativity* (Pearson, 2003).
